# Immunopathogenesis of Type 1 and Type 2 Leprosy Reaction: An Update Review

**DOI:** 10.7759/cureus.49155

**Published:** 2023-11-21

**Authors:** Dian Andriani Ratna Dewi, Christine Bella Putri Djatmiko, Indy Rachmawati, Nabila Arkania, Ni M Wiliantari, Farrasila Nadhira

**Affiliations:** 1 Department of Dermatovenereology, Faculty of Military Medicine, The Republic of Indonesia Defense University, Bogor, IDN; 2 Department of Dermatovenereology, Gatot Soebroto Central Army Hospital, Central Jakarta, IDN; 3 Department of Dermatovenereology, Faculty of Medicine, Public Health, and Nursing, Gadjah Mada University, Yogyakarta, IDN; 4 Department of Dermatovenereology, Ratna Dewi Principal Clinic, Bekasi, IDN

**Keywords:** nerve impairment, type 2, type 1, leprosy reaction, mycobacterium leprae, leprosy, immunopathogenesis

## Abstract

Leprosy reactions are acute exacerbations of the signs and symptoms of leprosy occurring during the natural course of the disease and during or after treatment. Left untreated or improperly managed, reactions can lead to severe nerve function impairment and subsequently to disabilities. In the present context of leprosy eradication efforts, leprosy reactions continue to pose a significant and enduring challenge. Type 1 leprosy reaction and type 2 leprosy reaction are substantial contributors to nerve impairment and the subsequent development of enduring impairments. The study of immunopathogenesis of leprosy reactions has emerged as a significant area of research due to its potential to identify critical targets for the early detection and management of these episodes. This study aims to reveal the pathogenesis of type 1 and 2 leprosy reactions so that they can form the basis for their treatment. The study used scientific journals from reputable platforms such as PubMed, Scopus, and Google Scholar to evaluate the pathogenesis of leprosy reaction type 1 and 2 in leprosy patients. This review indicates that the progression of leprosy nerve damage and sensitivity to reactions may be predicted using genetic and serum markers in the human host. A more profound comprehension of the molecular processes underlying leprosy reactions may offer a logical plan for early detection and leprosy reaction complication prevention.

## Introduction and background

Leprosy is a persistent granulomatous illness mainly attributed to two separate bacterial species, specifically *Mycobacterium leprae (M. leprae)* and *Mycobacterium lepromatosis (M. lepromatosis)*, with the latter being a more recently found causative agent. Both of these basil variants elicit similar pathogenic conditions in the stigma. The involvement of both sensory and motor nerves in the course of leprosy, both acute and chronic, is increasingly striking and has become the main component of this disease disorder. Permanent nerve damage can arise from the natural progression of untreated *M. leprae* infection or inflammatory reactions.

Pathophysiology of leprosy reaction

The main obstacle in providing care for leprosy patients is developing "reactions." The reactions listed above are caused by the intricate immune response to *M. leprae*, which might appear before, during, or after multi-drug treatment (MDT) administration. Two main classifications can be identified for leprosy reactions (LRs). Type 1 leprosy reaction (T1LR), commonly referred to as a "reversal" reaction, is classified as a type IV hypersensitivity reaction. The reaction described above is frequently found in individuals who have been diagnosed with borderline leprosy (BL). This condition is defined by a cellular immune response targeted explicitly towards specific antigens of the* M. leprae* bacteria [1]. The distinguishing characteristic of T1LR is the sudden onset of acute inflammation in preexisting cutaneous lesions or the development of new lesions, sometimes accompanied by neuritis [2].

Clinical manifestation and complication

Approximately 95% of cases involving T1LR are commonly identified during the first two years following MDT treatment [3]. The Type 2 leprosy reaction (T2LR) frequently manifests as erythema nodosum leprosum (ENL), an immune complex-mediated outcome of lepromatous leprosy (LL). The subcutaneous skin lesions display symptoms such as redness and pain, accompanied by fever and widespread inflammation that can affect various body parts, including the neurological system, eyes, joints, reproductive systems, and lymphatic tissues. Based on the research of Scollard et al., 1994 [1], it was found that there was no significant difference in the initial incidence of T2LR in patients who received MDT or not. However, de Oliveira et al., 2013 [4], found that the bulk of T2LR appears during the first year of MDT treatment. The probable consequences of a T2LR include long-lasting nerve dysfunction, physical deformity, and functional impairment [4]. The prevalence of nerve function impairment (NFI), which can be identified using clinical methods, is approximately 10% in individuals diagnosed with paucibacillary leprosy (PB) and 40% in individuals diagnosed with multibacillary leprosy (MB), particularly in those with T1LR. However, the prevalence of "silent neuropathy" caused by sub-clinical nerve involvement is widespread among the majority of patients afflicted with leprosy. This condition leads to damage in around 30% of nerve fibers before any sensory disruptions can be detected [5]. 

The occurrence of T1LR displays heterogeneity among various nations, with reported rates ranging from 19.7% to 30%, as shown in multiple prospective and retrospective studies. The occurrence of T2LR reactions in BL and LL individuals demonstrates notable variations across different geographical regions. Specifically, the frequencies of ENL reactions range from 19-26% in Asia to 37% in Brazil, as reported in a previous study. Exclusively in Mexico and Central America, the Lucio phenomenon is considered a globally restricted phenomenon. Lucio's phenomenon is a rare reactionary state seen in cases of diffuse lepromatous leprosy and assumed a form of a different type of leprosy reaction [6].

Despite extensive clinical and laboratory investigations, the underlying causes of leprosy reactions remain elusive, and their pathophysiological mechanisms need to be better understood. Numerous studies have yielded empirical data indicating a correlation between T1LR and the stimulation of the cellular immune system. Nevertheless, the precise stimulus for this activation remains unknown. In scholarly literature, T2LR is frequently acknowledged as an immunologically complex event. Nevertheless, considerable evidence suggests that circulating complexes may not be the key etiological factor. The existing findings are consistent with, although not conclusively implicating, complexes generated from tissues [7].

The assessment of physical disability in individuals impacted by leprosy functions as an epidemiological metric for evaluating the efficacy of leprosy initiatives, determining the timeliness of diagnosis (whether prompt or delayed), and tracking the advancement of patients during their treatment trajectory within healthcare institutions. In addition, the rapid identification of novel instances of leprosy and the administration of corticosteroid therapy for severe reactional episodes have the potential to prevent recurrent occurrences of irreversible nerve impairment [3]. Therefore, knowing the immunopathogenesis underlying the event of T1LR and T2LR can be the basis for preventing physical disability in leprosy patients.

Drawing from the context, we will explore the immunopathogenic mechanisms of T1LR and T2LR, as current scholarly investigations outline.

Search strategies

The approach used in this systematic review followed the instructions outlined in the Preferred Reporting Items for Systematic Reviews and Meta-Analysis (PRISMA) statement, which offers a standardized structure for reporting systematic reviews. A comprehensive review of the available literature was undertaken by employing many academic databases, including Scopus, PubMed, and Google Scholar. Search phrases were utilized to locate relevant scholarly works, including type 1 leprosy reaction, type 2 leprosy reaction, immunology, molecular mechanism, and randomized controlled trials (RCTs). The initial search was conducted in August 2023.

Inclusion criteria are the study that consists of the mechanism of T1LR and T2LR, the study with a randomized controlled trial study design, and a peer-reviewed article published between 2013-2023. Exclusion criteria are the study was a non-randomized controlled trial, an observational article, and a report that only describes T1LR or T2LR treatment.

Data extraction

The titles and abstracts were thoroughly examined to align with the predetermined inclusion criteria. The complete reports were evaluated to see whether the publications met the inclusion criteria, encompassing outcomes, interventions, research designs, and patient demographics. The rationales for the exclusion of studies were elucidated.

Quality assessment

**Figure 1:**
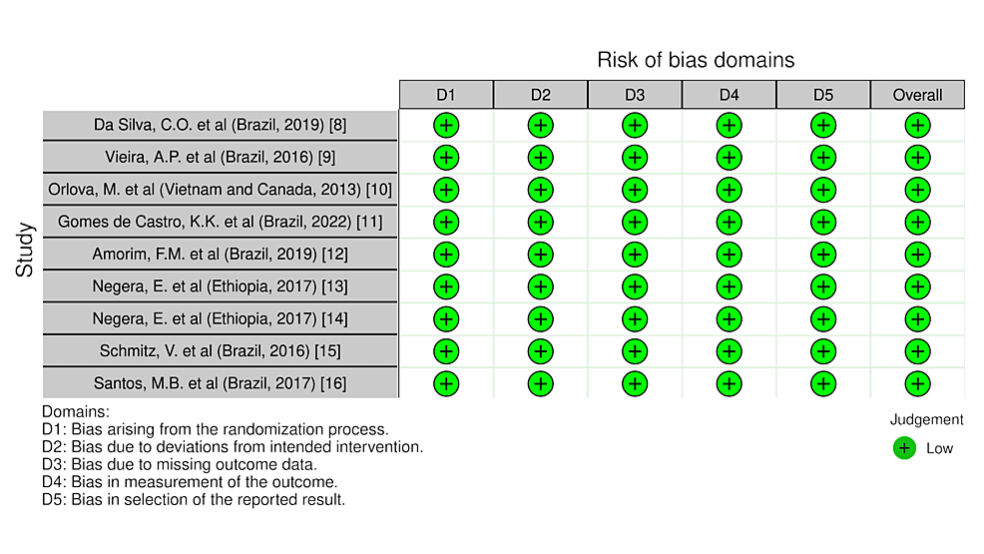
Quality assessment of selected studies using the Cochrane risk-of-bias technique for randomized trials, version 2. This figure was created by CB, one of the authors of this article.

The research's methodological quality was evaluated by two independent reviewers, CB and DARD, utilizing the Risk of Bias 2 (RoB2) methodologies. Cochrane Reviews primarily comprise randomized studies that have undergone evaluation for potential bias using the Cochrane risk-of-bias technique for randomized trials, version 2. The defined categories of bias in RoB2 cover a wide variety of possible issues with trials and their reporting. A series of inquiries known as "signaling questions" aims to obtain information regarding trial characteristics relevant to each domain's risk of bias. An algorithm proposes a bias risk estimate for each part based on the replies to the signaling questions. Bias risk ratings can vary from "Low" to "High," with "Some concerns" as an option (Figure 1).

Description of selected studies

**Figure 2:**
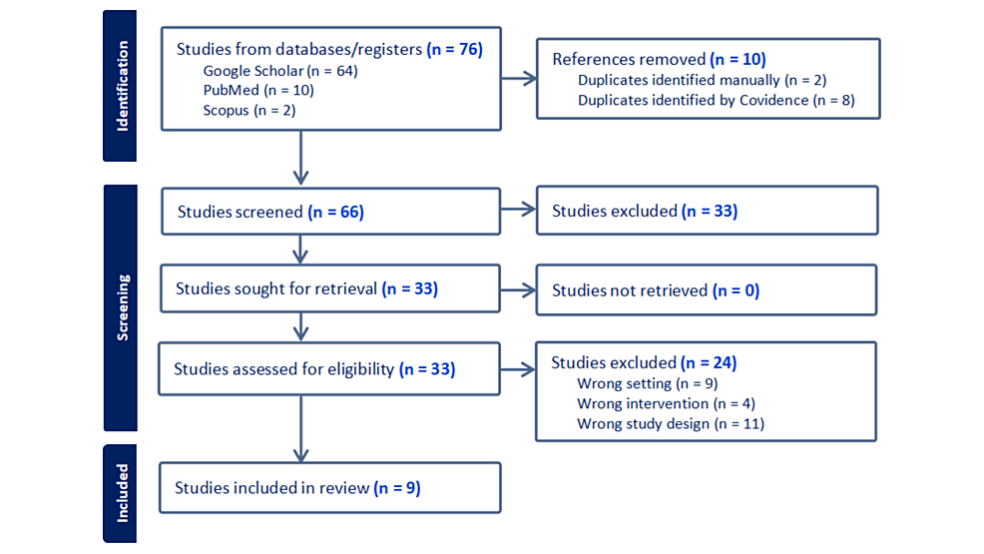
Preferred reporting items for systematic reviews and meta-analysis (PRISMA) flow sheet. This figure was created by CB, one of the authors of this article.

The systematic search showed 76 results. Only peer-reviewed English-language publications published between 2013 and 2023 were considered. The datasets underwent a process of removing a single instance of duplicated research. A comprehensive analysis was conducted on 66 articles, wherein their titles and abstracts were thoroughly examined. This process led to the further review of 31 full-text studies. Twenty-two studies were eliminated because they had the incorrect setting (n = 8), intervention (n = 2), research design (n = 11), and administration (n = 1). Nine studies were considered in this analysis (Figure 2, Table 1).

**Table 1:** Summary of selected study characteristics.

No.	Title	Author (Country where the research was carried out, year)	Aim of Study	Population	Results and Conclusions
1.	Neutrophil extracellular traps contribute to the pathogenesis of leprosy type 2 reaction.	Da Silva et al. (Brazil, 2019) [8]	To examine the production of neutrophil extracellular traps (NETs) in patients with type 2 leprosy reaction.	Forty individuals diagnosed with leprosy were categorized into two groups based on their leprosy reactions.	Both the whole-cell sonicate of *Mycobacterium leprae* and the CpG-Hlp complex, acting as a surrogate for a mycobacterial Toll-like receptor 9 (TLR9) ligand, have the potential to trigger the formation of neutrophil extracellular traps. There is a possible involvement of neutrophil extracellular traps in the development of type 2 leprosy reaction.
2.	Development of Type 2, But Not Type 1, Leprosy Reactions is Associated with a Severe Reduction of Circulating and In Situ Regulatory T-Cells	Vieira et al. (Brazil, 2016) [9]	To investigate the underlying mechanisms responsible for the reduction of Tregs in patients with type 2 leprosy reaction conditions.	Twenty-eight individuals exhibit clinically active and severe reactions.	Patients with type 2 leprosy reactions demonstrate notably reduced circulating and in situ quantities. Tregs were compared to both type 1 leprosy reaction patients and the control group. The observed decline was accompanied by a concurrent rise in interleukin-17 (IL-17) expression within the local environment while transforming growth factor-beta (TGF-β) expression experienced a decline.
3.	Gene Set Signature of Reversal Reaction Type I in Leprosy Patients	Orlova et al. (Vietnam and Canada, 2013) [10]	To identify the type 1 leprosy reaction gene set and assess as well as validate the findings using a retrospective design.	A cohort of 43 persons was recently diagnosed with borderline leprosy without type 1 leprosy reaction.	The results suggest a role for intrinsic factors in type 1 leprosy reaction, which is a first step toward establishing a prognostic genetic profile for type 1 leprosy reaction.
4.	Downmodulation of Regulatory T Cells Producing TGF-β Participates in Pathogenesis of Leprosy Reactions	De Castro et al. (Brazil, 2022) [11]	To conduct a phenotypic and functional analysis of CD4^+^ and CD8^+^ Treg cells both in their ex vivo state and in response to *Mycobacterium leprae*.	Twenty-two people were divided into three groups. Eighteen newly diagnosed and untreated individuals Multibacillary. Nineteen individuals had undergone type 1 leprosy or type 2 leprosy reaction episodes of reactional Multibacillary, and 15 healthy volunteers served as controls.	A reduction in the populations of CD4^+ ^transforming growth factor-beta (TGF-β^+^) Tregs and CD8^+^transforming growth factor-beta (TGF-β^+^) Tregs has been seen in persons affected with Multibacillary during both types of reactional episodes. Alterations in the cytokine profile were additionally observed in type 2 leprosy reaction., concomitant with an elevation in the concentrations of interleukin-17 (IL-17) and interleukin-6 (IL-6) in the supernatant.
5.	Differential immunoglobulin and complement levels in leprosy prior to the development of reversal reaction and erythema nodosum leprosum	Amorim et al. (Brazil, 2019) [12]	This study looks into the biological variables influencing differences in gene expression within the canonical pathways connected to complement and immunoglobulin in the setting of immune responses to leprosy.	The population size is 96 with aged 18 years and above	The presence of reduced C4 levels and increased anti-*Mycobacterium leprae* antibodies among individuals newly diagnosed with leprosy may serve as risk factors for the eventual occurrence of leprosy immunological reactions.
6.	T-cell regulation in Erythema Nodosum Leprosum	Negera et al. (Ethiopia, 2017) [13]	To examine the Treg-cells in individuals diagnosed with erythema nodosum leprosum, a comprehensive investigation was conducted.	A cohort of 46 individuals exhibiting erythema nodosum leprosum reaction and 31 individuals with non-reactional Lepromatous-leprosy were selected.	There is a correlation between the presence of erythema nodosum leprosum and a reduction in the proportion of Treg-cells, as well as an increase in the CD4^+^ to CD8^+^ T-cell ratio. Additionally, there is an observed rise in the amount of interleukin-17 (IL-17)-producing T-cells. The initiation erythema nodosum leprosum reactions can be attributed to dysregulation of the immune system.
7.	Increased activated memory B-cells in the peripheral blood of patients with erythema nodosum leprosum reactions	Negera et al. (Ethiopia, 2017) [14]	To investigate the role of B-cells in the development of erythema nodosum leprosum, it is imperative to conduct a thorough analysis.	Patients diagnosed with erythema nodosum leprosum were administered a course of steroid treatment. Multi-drug therapy was administered to all individuals diagnosed with leprosy. The total number of patients was 60.	Patients who did not receive treatment for erythema nodosum leprosum exhibited a greater abundance of B-cells that had encountered antigens and were in an activated state, as compared to persons who did not have this reaction, specifically Lepromatous-leprosy patients. This observation implies a connection between B-cells and the pathogenesis of erythema nodosum leprosum.
8.	Expression of CD64 on Circulating Neutrophils Favoring Systemic Inflammatory Status in Erythema Nodosum Leprosum	Schmitz et al. (Brazil, 2016) [15]	To examine the expression of CD64 during erythema nodosum leprosum and determine whether thalidomide medication had any effect on its expression.	In Group I, there were a total of 16 health volunteers, consisting of 7 females and nine males. Group II consisted of 62 individuals diagnosed with leprosy, comprising 46 males and 16 females. The total number of patients in the study was 78.	There was a positive correlation observed between the severity of erythema nodosum leprosum symptoms and the level of CD64 expression. The potential utility of CD64 expression in neutrophils as a predictive biomarker for erythema nodosum leprosum is noteworthy. Furthermore, assessing the CD64 response could offer valuable insights into the severity of this particular medical issue.
9.	Distinct Roles of Th17 and Th1 Cells in Inflammatory Responses Associated with the Presentation of Paucibacillary Leprosy and Leprosy Reactions	Santos et al. (Brazil, 2017) [16]	To comprehensively examine the inflammatory cytokine profile linked to the various clinical manifestations of leprosy, a unified approach is employed.	A total of 74 patients were identified who had symptoms of leprosy, leprosy responses, and neurological impairments. Each individual possessed a scar that served as evidence of a previous Bacillus Calmette-Guérin (BCG) immunization.	T-helper-17 cells significantly facilitate a robust inflammatory response, ultimately developing paucibacillary forms in individuals affected with leprosy. Nevertheless, these cells do not function as a prognostic indicator for the eventual manifestation of inflammatory leprosy reactions in patients with multibacillary. This suggests that T-helper-17 cells provide immunity against *Mycobacterium leprae* infection and play a role in the progression of milder clinical symptoms in individuals with leprosy.

## Review

The immunopathogenesis of leprosy reaction

The persistent occurrence of reactions in individuals affected by leprosy is a significant obstacle to neurological problems associated with leprosy, even with the successful eradication of the disease. This phenomenon is primarily associated with the onset of nerve dysfunction and physical impairment. Considering the potential for extended latency, it is worth mentioning that LRs can manifest themselves for several months or even years following the conclusion of MDT. Hence, it can be anticipated that the resultant impairment will persist, even in the highly improbable event of total elimination of leprosy. Cohort studies have yielded estimations suggesting that the prevalence of disability among individuals afflicted with leprosy ranges from 16% to 56%. This impairment is primarily ascribed to the occurrence of reactional episodes. Based on a comprehensive study from Nery et al., it has been determined that individuals diagnosed with T1LR may still encounter a notable incidence of enduring peripheral nerve impairment, affecting approximately 40% of the affected population [17].

The research by van Brakel et al. involved evaluating individuals presenting with neuritis or reactional episodes. The researchers employed nerve conduction investigations and quantitative sensory testing as diagnostic tools. The findings indicated that individuals, whether experiencing these conditions independently or concurrently, exhibited subclinical manifestations of neuropathy up to 12 weeks before the emergence of any observable pathological changes in their health status. The finding suggests the possibility of early detection and intervention to mitigate the advancement of nerve damage and abnormalities. In the current clinical context, it is imperative to establish reliable laboratory tests that can aid in promptly identifying LRs and assessing treatment efficacy. The initiation of treatment for LRs is primarily administered following a clinical diagnosis [18].

**Figure 3:**
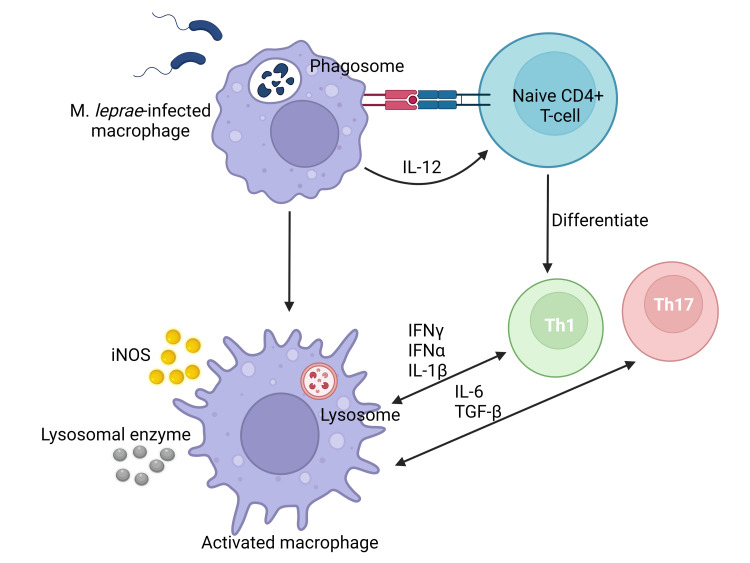
The underlying pathophysiological process of the type 1 leprosy reaction predominantly achieved by coordinating CD4+ T Cells immunological response. Activated macrophages induce the secretion of pro-inflammatory T-helper 1 cytokines such as interferon-gamma (IFN-γ), tumor necrosis factor-alpha (TNF-α), interleukin-1 beta (IL-1β), interleukin-6 (IL-6), interleukin-2 (IL-2), interleukin-12 (IL-12), transforming growth factor-beta (TGF-β), and inducible nitric oxide synthase (iNOS). These molecules activate immune cells, the initiation of inflammatory responses, and the modulation of immunological reactions. The cytokines indicated above have a crucial impact on the promotion of tissue damage. This figure was created by CB, one of the authors of this article, with Biorender.com.

The occurrence of a leprosy reaction can be attributed to the immunological response of the immune system towards* M. leprae*, and it contributes to the development of neurological problems associated with leprosy. This syndrome arises due to an abrupt and robust immune reaction to the deteriorated constituents of the leprosy bacteria. The phenomenon of interest is frequently noticed after the implementation of antibiotic treatment. The occurrence of this specific reaction has been observed in around 30 to 50% of persons who have been clinically diagnosed with leprosy [7]. T1LR, alternatively referred to as a borderline reaction or "reversal" reaction, manifests in individuals classified as borderline (BT, BB, and BL) as well as in certain cases of LL. The coexistence of neuroinflammation alongside acute inflammation of a preexisting cutaneous lesion is a distinctive hallmark of T1LR leukocyte recruitment. Over 95% of T1LR cases tend to present either during the initial diagnosis of leprosy or within the initial two-year period of MDT [19]. Approximately 10% of people with paucibacillary and 40% of people with multibacillary have neurological impairment. Neurological impairment is more common in those with T1LR. Type 1 leprosy reaction is the primary etiology behind the persistent nerve damage, physical deformities, and substantial functional disability observed in individuals afflicted with *M. leprae* infection [20]. The pathophysiology of T1LR is characterized by an immune response marked by a type-IV hypersensitivity reaction [21]. The immune-mediated tissue damage noticed in T1LR may be a result of CD4^+^ T cell infiltration [22]. Th1 cells are responsible for the production of a diverse array of cytokines, including tumor necrosis factor α (TNFα), interleukin (IL)-2, IL-1β, and interferon‐gamma (IFN‐γ). The cytokines play pivotal roles within the framework of T1LR (Figure 3).

**Figure 4:**
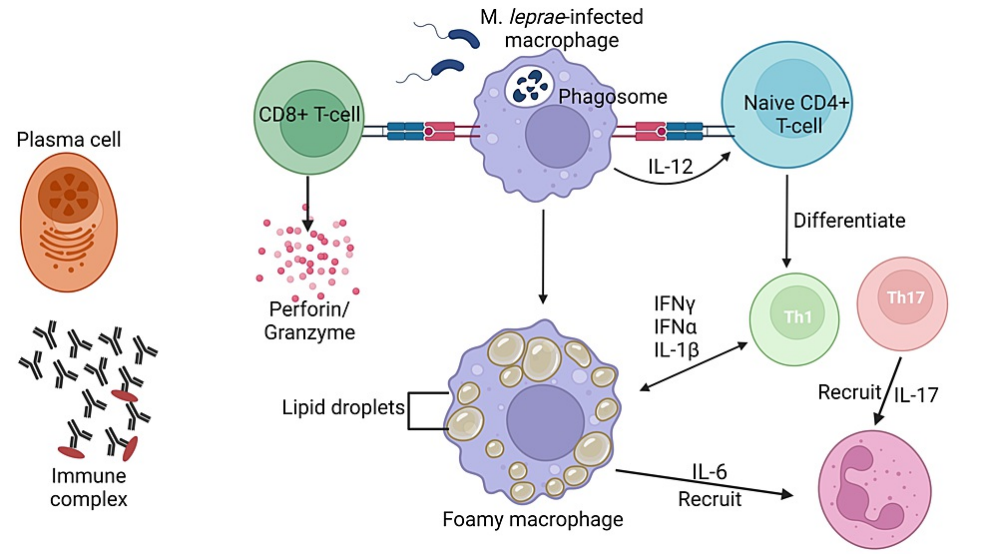
Erythema nodosum leprosum is a pathological condition characterized by a robust inflammatory response involving the recruitment of neutrophils. The detection of complement activation, immune complexes, increased CD4+/CD8+ T cell subset ratios, and a notable concentration of tumor necrosis factor-alpha (TNF-α) can be observed in both the lesions and the systemic circulation. There is a surplus of B lymphocytes and plasma cells, which exhibit the capacity to produce antibodies. CD8+ T lymphocytes secrete a repertoire of cytotoxic granule proteins, such as perforin and granzymes, that elicit apoptosis in target cells. IFN-α: interferon-alpha, IFN-γ: interferon‐gamma, IL: interleukin This figure was created by CB, one of the authors of this article, with Biorender.com.

ENL is a dermatological condition distinguished by the infiltration of neutrophils and the manifestation of systemic inflammation. The observed lesions and circulation demonstrate the complement system's activation, leading to immune complexes production and an elevated ratio of CD4^+^/CD8^+^ T cell subsets, and there were higher levels of TNF-α, a pro-inflammatory cytokine [23]. In the acute phase of ENL, there is a notable presence of neutrophilic infiltration observed within the lesions. Type III hypersensitivity reactions, sometimes called immune complex-mediated hypersensitivity reactions, can occur due to the presence of antibodies that create immune complexes within tissues and blood vessels [24] (Figure 4).

ENL manifests in more than 50% of LL cases and approximately 5-10% of instances of BL. The immune response elucidated in this particular context is intricately linked with TNFα, a cytokine produced in response to either *M. leprae* itself or the inactivated constituents of the bacilli subsequent to the administration of antimicrobial therapy [25]. The majority of patients are young, male, and have lepromatous leprosy and a high mean of Bacterial Index. Many individuals have chronic and severe diseases because individuals have acute EN; a portion of these will continue to experience ENL for longer than six months and, therefore, be reclassified as recurrent or chronic. Chronic ENL is significantly more likely to be classified as severe than acute or recurrent ENL. The cardinal presentation is characterized by a dolorous and often tender erythematous cutaneous lesion within the subcutaneous region. Moreover, it is imperative to acknowledge that systemic inflammation can influence diverse anatomical structures, including nerves, joints, ocular structures, gonads, and lymph nodes, occasionally concomitant with increased body temperature [25].

Factors that influence the immunopathogenesis of leprosy reactions

The interaction between host immune response and infection is an ideal model for studying the immune-related spectrum in leprosy. Leprosy is, to some extent, a genetic disease because the genetic factors of hosts have long been considered significant contributors to this disease. We attempted to link factors influencing leprosy reactions by reviewing recent research examining immunological and genetic factors.

Agent Virulence Factors

Patients diagnosed with T1LR exhibit the *M. leprae* antigens within Schwann cells and macrophages, as substantiated by detecting these antigens in the nerves and skin [26]. A research study conducted in Brazil investigated the correlation between the presence of *M. leprae* DNA in cutaneous lesions and the occurrence of T1LR in patients diagnosed with slit-skin smear-negative, single lesions, and pacibacillary. The research findings elucidated that subjects who demonstrated *M. leprae* DNA in their cutaneous lesions were predisposed to the occurrence of T1LR when compared with individuals lacking detectable *M. leprae* DNA [27].

Innate Immunity

Patients with leprosy experience reactional episodes ultimately decided by local innate immune processes. The functions performed by neutrophils play a critical part in the interaction between the host and mycobacteria, extending beyond their role as migratory cells that respond to chemoattractants specifically in leprosy. Furthermore, there needs to be a more comprehensive understanding regarding the phenotypic and functional attributes of macrophages residing in the skin and their intricate interactions with the sensory nerve system within the skin [28].

Approximately half of the individuals who receive a diagnosis of the multibacillary variant of leprosy are anticipated to experience T2LR. The reactions are characterized by periods of acute systemic inflammation, which can potentially worsen the individuals' clinical condition. Further investigation is warranted into the etiology of T2LR and the immunoinflammatory cascades involved in its pathogenesis. The capacity of the host to counteract the invasion of pathogens is a crucial aspect of the innate immune system. The presence of pattern recognition receptors (PRRs) can be partially attributable to this biological process. These receptors demonstrate a remarkable capacity to discriminate and recognize a diverse array of microbial ligands, enhancing the process's overall efficacy. The identification of mycobacterial lipoproteins has been conclusively demonstrated to involve Toll-like receptor 2 (TLR2). Activation of Toll-like receptors (TLRs) results in the subsequent activation of nuclear factor-kappa B (NF-kB). The transcription factor plays a pivotal role in regulating transcription for a multitude of genes crucial to immune responses [29].

The latest discovery linking T2LR cases with innate immunity we reviewed from the articles we extracted was research conducted by da Silva et al., 2019 [8]. The study has elucidated the role of Toll-like receptor 9 (TLR9) in recognizing nucleic acids, thereby serving as a vital mechanism for activating innate immunity during the T2LR. TLR9 is a DNA-sensing receptor expressed in professional innate immune cells such as DCs and macrophages. Neutrophil extracellular traps (NETs) release induction by TLR9 ligand. The significance of neutrophil DNA extracellular traps as a crucial reservoir of endogenous DNA within this specific milieu has been firmly established [8].

Other Genetic Factors

Research on the relationship between genetic factors and leprosy infection was carried out by Zhang et al., 2009 [30]. They conducted a two-stage genome-wide association study by genotyping 706 patients and 1,225 controls using the Human610-Quad BeadChip (Illumina, San Diego, CA, USA). They then tested three independent replication sets for the association between the presence of leprosy and the 93 single-nucleotide polymorphisms (SNPs) most closely associated with the disease in genome-wide association studies. Together, these replication sets consisted of 3,254 patients and 5,955 controls. They also tested for heterogeneity of association (or lack thereof) between 93 SNPs and disease, stratified by clinical subtype (multibacillary vs. paucibacillary). This study concludes that variants of genes in the NOD2-mediated signaling pathway (which regulates the innate immune response) are associated with susceptibility to infection with *M. leprae*.

The latest research shows the relationship between LRs and genetic factors by Orlova et al. 2013 [10]. They undertook a retrospective investigation to evaluate the intrinsic heterogeneity in the immune reaction to *M. leprae* among individuals in good health and those previously suffering from leprosy, with or without T1LR. Upon conducting a retrospective analysis, the researchers observed a significant discrepancy in the expression of 581 genes following exposure of whole blood to *M. leprae* sonicate. The gene set signature encompasses 44 genes derived from the T1LR gene family, demonstrating distinctive regulation patterns. This observation suggests a fundamental dysfunction in regulating the immune response towards antigens originating from *M. leprae*. Identifying the T1LR gene set signifies a crucial primary phase in establishing a genetic profile for individuals afflicted by leprosy, distinguished by an augmented vulnerability to T1LR and consequent nerve dysfunction [10].

Leprosy is related to a range of genes implicated in the differentiation and responses of Th1, Th2, and Th17 cells. The downstream cascade of IL-23 signaling is the Janus kinase (JAK)-signal transducer and activator of the transcription (STAT) signaling system, and two important members of this pathway, TYK2 and SOCS1, have also been linked to leprosy. The results of this study indicate the potential role of Th17 responses in the pathogenesis of leprosy [10].

Adaptive Immunity

Updated review of the immunopathogenesis of leprosy reactions in terms of adaptive immunity factors based on research included in the characteristics of this review are:

Based on research conducted by Viera et al., 2016 [9], the postulate was put forward that the dysregulation of regulatory T cells (Tregs) may contribute to the pathogenesis of leprosy manifestations. The researchers investigated the prevalence of circulating Tregs in individuals diagnosed with T1LR and T2LR. The investigation also assessed Tregs and cells exhibiting IL-17, IL-6, and transforming growth factor beta (TGF-β) at the specific anatomical location. Patients presenting with T2LR showed a notable decrease in the number of Tregs in the bloodstream and at the site of inflammation compared to patients experiencing T1LR and individuals unaffected by this medical condition (controls). The observed reduction in levels was accompanied by an increase in the expression of IL-17 within the tissue, while a decrease in the expression of TGF-β was evidenced. The study findings indicate that there was no statistically significant disparity in the quantities of forkhead box protein P3+ (FoxP3+) and IL-17^+^ cells detected in biopsies obtained from individuals diagnosed with T1LR and T2LR before the initiation of response episodes [9].

However, it is important that in the biopsies acquired during the immune response, individuals with T2LR demonstrated a decrease in the population of Tregs and a concurrent increase in the presence of IL-17^+^ cells. On the contrary, individuals diagnosed with T1LR exhibited a contrasting trend: a notable augmentation in Tregs alongside a decline in IL-17^+^ cells. Furthermore, the investigators noted a decrease in the proliferation of Tregs upon stimulation with *M. leprae* in an experimental laboratory environment. Again, an intriguing observation was made regarding a downward trend in the expression levels of FoxP3 and the immunosuppressive molecule cytotoxic T-lymphocyte-associated protein 4 (CTLA-4) within the T2LR Treg population. The authors present compelling evidence to substantiate that in T2LR, the reduction in Tregs may foster the emergence of T-helper-17 responses, characteristic of this specific immune reaction [9].

de Castro et al. (2022) conducted a comprehensive investigation of cellular specimens procured from 18 patients with untreated multibacillary, 19 patients manifesting reactional multibacillary (experiencing either T1LR or T2LR), and 15 healthy volunteers who were enlisted as control subjects. The enzyme-linked immunosorbent assay (ELISA) was employed to examine the production of cytokines in the supernatant acquired from cultures of peripheral blood mononuclear cells following exposure to *M. leprae*. The result of the study revealed the presence of Treg cells, which exhibit the expression of CD4^+^TGF-β^+^ and CD8^+^TGF-β^+^ in individuals afflicted with multibacillary disease. This observation was correlated with an aberration in the concentrations of pro-inflammatory cytokines. This study offers innovative perspectives on the pathophysiological mechanisms underlying leprosy responses, focusing on the role of CD8^+^ Tregs. The subset of cellular entities has been subject to inadequate scrutiny in prior investigations, notwithstanding its noteworthy implications for the clinical advancement of leprosy [11].

In a study conducted by Amorim et al. (2019), it was demonstrated that individuals diagnosed with multibacillary exhibited a diminished prevalence of CD32B in plasmablasts. The potential contribution of a diminished presence of negative feedback mechanisms to the increased synthesis of antibodies is worth considering. In individuals with intact immune competence, the interaction of CD32B on B cells has been noted to exert an inhibitory effect on the production of antibodies and attenuate the antibody-independent functions of B cells. The observed decrease in CD32B expression may enhance antibody production and other immune responses that are not directly related to antibody production. The investigators postulate that a dysregulation in the modulation of CD32 could contribute to the protracted persistence of *M. leprae* infection. The imbalance gives rise to a heightened yet insufficient humoral response in regulating bacterial proliferation, along with an escalated immunological reaction manifested as T1LR and T2LR [12]. The observed variations in expressions of co-receptors and the levels of immunoglobulins before and during immunological reaction indicate a notable participation of humoral immunity in the development of T1LR and T2LR. Individuals recently diagnosed with leprosy exhibit diminished levels of C4 and elevated levels of anti-*M. leprae *antibodies may present an increased risk for the subsequent development of leprosy immunological reactions. However, around 33% exhibit immunological reactions even when the patients are administered antibiotics. The study unveiled a notable association between concentrations of antibodies targeting *M. leprae* from leprosy identification time probability of encountering immunological reactions within the subsequent two years. Furthermore, assessing C4 levels in the circulatory system may serve as a valuable modality for monitoring the progression and development of leprosy reactions [12]. 

Before de Castro et al. (2020) [11] studied the role of Tregs on T2LR, Negera et al. (2017) [13] conducted a comprehensive investigation of the Treg cells in individuals diagnosed with erythema nodosum leprosum. A cohort research of 46 individuals exhibiting erythema nodosum leprosum reaction and 31 individuals with non-reactional lepromatous leprosy were selected. There is a correlation between the presence of T2LR and a reduction in the proportion of Treg cells, as well as an increase in the CD4^+^ to CD8^+^ T cell ratio. Additionally, there is an observed rise in the amount of interleukin-17 (IL-17)-producing T cells. The initiation of erythema nodosum leprosum reactions can be attributed to dysregulation of the immune system, T2LR is associated with loss of T cell regulation. It has shown that Tregs may protect from non-specific memory T cell activation and potential tissue damage. Hence, the reduced frequency ofCD4^+^Tregs and the increased CD4^+^/CD8^+^ T cells ratio in untreated T2LR patients may explain the possibility of induction of excessive immune activation owning to the pre-existing high load of bacterial antigens in patients with lepromatous leprosy. This immune imbalance could lead to the initiation of T2LR either by permitting increased production of antibodies critical to immune-complex formation or as a cell-mediated immune response in patients with lepromatous leprosy [13].

Negera et al. (2017) [14] continued their research to investigate the role of B cells in the development of ENL, it is very important to carry out a thorough analysis. Patients diagnosed with ENL are given steroid treatment. Multi-drug therapy is given to all individuals diagnosed with leprosy. The total number of patients was 60. Patients who did not receive treatment for ENL showed more B cells that had encountered the antigen and were in an activated state, compared with people who did not experience this reaction, especially in the LL type of leprosy. The immunological dysregulation observed in individuals diagnosed with LL type may reduce the significant burden of bacterial antigens within their system. These observations imply a link between B cells and the pathogenesis of ENL. This phenomenon may arise through the augmentation of antibody production, vital for forming immune complexes, or by stimulating the cellular immune system [14].

The exact function of neutrophils in ENL remains ambiguous. Schmitz et al. (2016) [15] examined the expression of CD64 during ENL and determined whether thalidomide medication had any effect on its expression. The research findings have elucidated a correlation between the presence of CD64-expressing neutrophils and the manifestation of systemic inflammation in individuals afflicted with ENL. Analyses of circulating neutrophils revealed that ENL patients expressed higher levels of surface CD64 in comparison to those with non-reactional leprosy and that the severity of ENL was coupled with high levels of CD64 expression. As a result, it is conceivable to speculate that the level of CD64 expression seen in peripheral blood neutrophils may serve as an accurate indicator of the severity of ENL. Despite the limited number of patients included in this study, it demonstrated that measurement of neutrophil CD64 expression could be used as a prognostic biomarker of ENL and that quantifying the CD64 response could also help indicate the severity of ENL. These patients exhibit compromised cellular immunity yet retain adequate quantities of B lymphocytes and plasma cells, enabling them to generate antibodies targeting *M. leprae*. Indeed, the methodology adopted found that circulating neutrophil CD64 expression could provide a rapid and non-invasive ENL diagnosis capable of detecting reactions in outpatient clinics as well as leprosy reference centers, leading to more effective therapeutic decisions. Therefore, this significant finding lays the groundwork for subsequent inquiries that specifically address B cells as a therapeutic target for developing effective pharmaceutical interventions in treating ENL [15].

The study conducted by Santos et al. (2017) elucidated that lesions acquired from individuals diagnosed with tuberculoid leprosy (TT) showed an augmented abundance of CD4^+^ IL-17A^+^ cells in comparison to lesions obtained from individuals diagnosed with LL. Patients with paucibacillary leprosy serum exhibited elevated levels of IL-17A and IL-1b compared to individuals with multibacillary. The serum concentrations of IFN-γ that showed the utmost levels were noted in the blood samples of individuals diagnosed with multibacillary, specifically those who manifested leprosy reactions. In aggregate, these observations imply a correlation between Th1 cells and the manifestation of symptoms in leprosy. In contrast, it has been observed that Th17 cells exhibit a significant correlation with a robust inflammatory response in individuals diagnosed with paucibacillary. However, the authors failed to provide prognostic value regarding leprosy reactions in patients diagnosed with multibacillary. This observation suggests that Th17 cells have a protective function in the context of *M. leprae* infection and are involved in the progression of milder clinical presentations of leprosy [16].

Immunopathogenesis of nerve damage

Deformity and disability in leprosy are caused by impaired nerve function. From the literature reviewed, it was found that nerve damage can be detected long before it becomes clinical. These new findings may have important implications for treatment because steroid therapy for clinically detected neurological dysfunction is unsatisfactory. Leprosy neuritis widely differs based on the type of cellular inflammation of nerves and the type of leprosy.

Neuritis on T1LR is characterized by neuropathic disorders manifesting in dermal nerve terminals, subcutaneous nerve structures, and nerve trunks. Epithelioid granulomas result in compression that damages nerve fibers. The observed pathological findings relate to the convergence of the immunological cascade and biomechanical influences [31]. 

Peripheral nerve injury is a manifestation commonly observed in leprosy patients with T1LR. The etiology of the damage of Schwann cells is postulated as a result of harm during the inflammatory process. Activation of TNF-α, CD4^+^ cytolytic T lymphocytes, and TGF-β caused significant detachment and lysis of Schwann cells [32].

**Figure 5:**
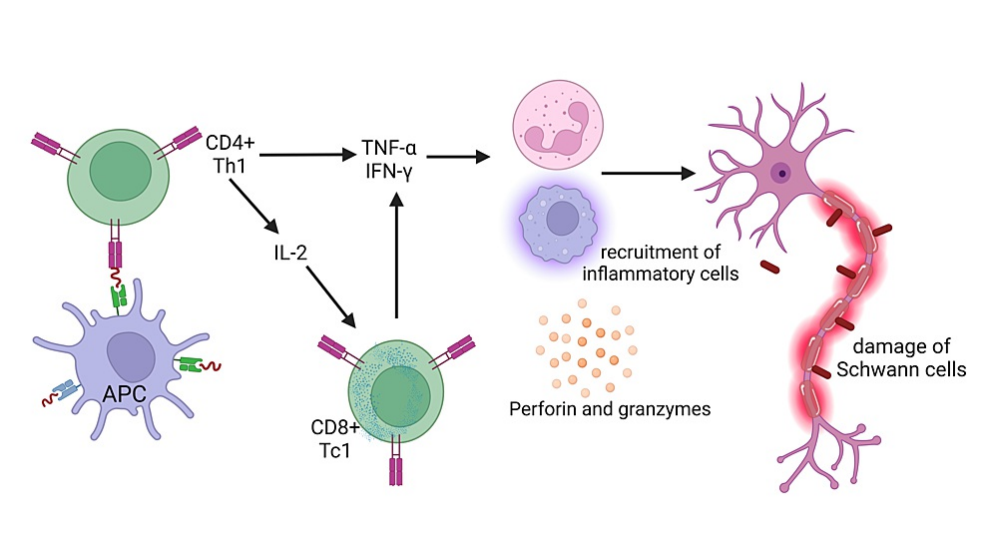
The mechanism of nerve damage in type 1 leprosy reaction. Activated macrophages release pro-inflammatory cytokines, such as tumor necrosis factor-alpha (TNF-α), interleukin-1 beta (IL-1β), and interleukin-6 (IL-6). The cytokines caused increasing endothelial barrier permeability, thereby promoting immune cell transmigration towards the inflammatory locus. As a result, this series of physiological processes ultimately manifests localized edema, erythema, and increased temperature. Furthermore, it is essential to highlight that macrophages can release lysosomal enzymes, complement components, and reactive oxygen species in a localized anatomical area. The complex mechanism has a crucial role in the progression of tissue damage. Perforin facilitates pore formation within the target cell's membrane and promotes the entry of granzymes into the cellular environment, thus initiating the complex series of apoptosis. APC: antigen-presenting cell, IFN‐γ: interferon‐gamma, IL: interleukin This figure was created by NA, one of the authors of this article, with Biorender.com.

One potential immunopathogenic mechanism contributing to the destruction of Schwann cells and nerves in leprosy is the capacity of infected Schwann cells to process and transport antigens originating from* M. leprae* to antigen-specific, inflammatory type 1 T cells. These T cells are responsible for inducing harm and lysis of the infected Schwann cells. While CD8 and CD4 cytotoxic T cells are involved in this particular mechanism, CD4^+^ T cells have significant relevance. There is an increase of CD4^+^ T lymphocytes within the granuloma centers of leprosy patients with T1LR (Figure 5) [33].

The pathophysiological mechanisms that contribute to the development of ENL involve the participation of immune complexes and type III hypersensitivity reactions. Type III hypersensitivity, called immune complex-mediated hypersensitivity, arises from inadequate elimination of immune complexes formed by binding antigens and antibodies. This results in an inflammatory reaction and the mobilization of leukocytes. ENL patients exhibit the clinical manifestation of circulating immune complexes that specifically target phenolic glycolipid-1 (PGL-1) and the primary cytosolic protein of *M. leprae.* Nevertheless, the precise mechanism by which immune complexes contribute to the development of ENL still needs to be fully understood. Neutrophils are of utmost importance in the initial stages of leprosy pathogenesis because they have the function of phagocytosis. This process involves engulfing the *M. leprae*, followed by the subsequent release of pro-inflammatory mediators [34].

**Figure 6:**
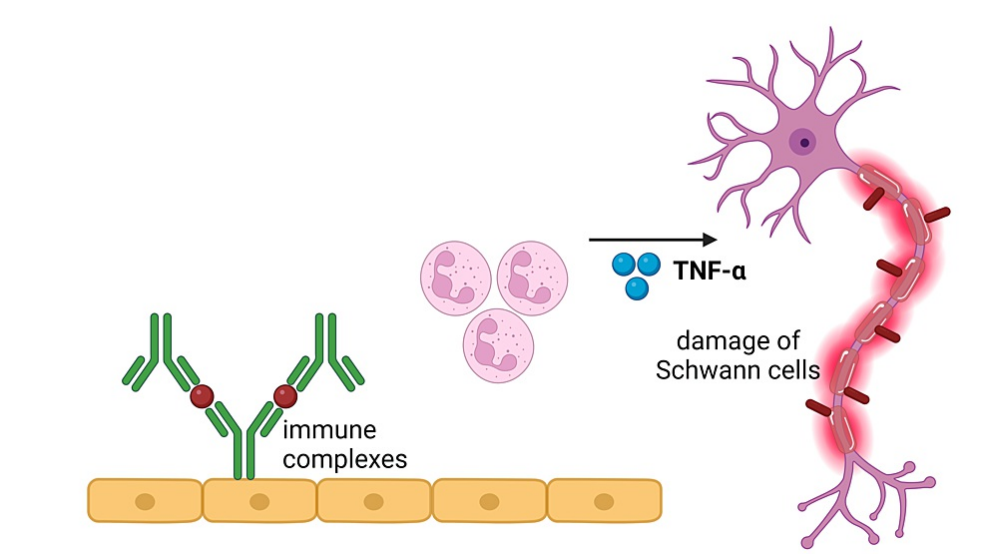
Mechanism of nerve damage caused by type 2 leprosy reaction. The mechanisms underlying the degradation of Schwann cells lead to subsequent nerve damage in individuals afflicted with leprosy. Neutrophils play a pivotal role in the pathogenesis of erythema nodosum leprosum by generating a substantial amount of tumor necrosis factor-alpha (TNF-α) and interleukin-8 (IL-8), which are closely linked to the induction of tissue damage. This observation is consistent with the established role of CD64 in promoting the synthesis of pro-inflammatory cytokines. This figure was created by FN, one of the authors of this article, with Biorender.com.

The investigation of innate immune response in nerve injury by observing Toll-like receptor 2 (TLR2) has also been documented in Schwann cells. In the leprosy lesions, Schwann cells demonstrating TLR2 expression undergo programmed cell death or apoptosis. This phenomenon can contribute to nerve impairment among individuals suffering from leprosy [35]. Nerve injury may manifest in the absence of apoptosis or lysis. This phenomenon is caused by demyelination from exposure to *M. leprae* without immune cells (Figure 6) [36].

## Conclusions

The factors influencing leprosy reaction conditions are still not fully known. Various studies have attempted to uncover various backgrounds that underlie leprosy reactions. Several studies have attempted to reveal the immunopathogenesis of leprosy reactions in the last 10 years. Several researchers have uncovered new things regarding the involvement of innate immunity, genetic factors, and adaptive immunity.

Type 1 leprosy reaction, also referred to as a type IV hypersensitivity reaction, is an immunological response mediated by the innate immune system. This reaction, known as delayed-type hypersensitivity, is characterized by a spontaneous occurrence in the presence of *M. leprae*. This condition is characterized by a gradual shift towards the tuberculoid end of the disease spectrum, concomitant with reducing the number of bacilli, strong reactivity in skin tests, increased responsiveness of lymphocytes, and a predominant Th1 immune response. Regarding the involvement of innate immunity, recent research reveals the role of Toll-like receptor 9 in recognizing nucleic acids in T2LR, which induces the release of NETs. 

Several researchers also revealed the relationship between leprosy reaction and genetic factors. They found that the T1LR gene set suggests a fundamental dysfunction in regulating the immune response towards antigens originating from *M. leprae,* distinguished by an augmented susceptibility to T1LR and consequent nerve dysfunction and the biological variables influencing differences in gene expression within the canonical pathways connected to complement and immunoglobulin in the setting of immune responses to leprosy.

Several researchers suggest that the dysregulation of Tregs may contribute to the pathogenesis of leprosy manifestations. The research results in innovative perspectives on the pathophysiological mechanisms underlying leprosy reactions, focusing on the role of CD8+ Tregs. There is a correlation between the presence of T2LR and a reduction in the proportion of Treg-cells and an increase in the CD4+ to CD8+ T-cell ratio. There is also a link between B cells and the pathogenesis of ENL. This phenomenon may arise through the augmentation of antibody production, vital for forming immune complexes, or by stimulating the cellular immune system.

The recent study also found that the level of CD64 expression seen in peripheral blood neutrophils may serve as an accurate indicator of the severity of ENL. Another recent study found the implication of a correlation between Th1 cells and the manifestation of symptoms in leprosy. In contrast, it has been observed that Th17 cells exhibit a significant correlation with a robust inflammatory response in individuals diagnosed with paucibacillary. This observation suggests that Th17 cells have a protective function in *M. leprae* infection and are involved in the progression of milder clinical presentations of leprosy.

Even though there are different mechanisms of immunopathogenesis, both T1LR and T2LR can cause nerve damage in leprosy patients. By understanding the immunopathogenesis of leprosy reactions, doctors can provide therapy according to the underlying mechanism to prevent further nerve damage.
